# Language combinations of multilinguals are reflected in their first-language knowledge and processing

**DOI:** 10.1038/s41598-023-27952-2

**Published:** 2023-02-02

**Authors:** Olga Kepinska, Jocelyn Caballero, Myriam Oliver, Rebecca A. Marks, Stephanie L. Haft, Leo Zekelman, Ioulia Kovelman, Yuuko Uchikoshi, Fumiko Hoeft

**Affiliations:** 1 Department of Psychiatry and Behavioral Sciences, Weill Institute for Neurosciences, University of California, San Francisco, San Francisco, CA 94143 USA; 2 Department of Psychological Sciences, University of Connecticut, Storrs, CT 06269 USA; 3 Brain and Language Lab, Cognitive Science Hub, University of Vienna, 1090 Vienna, Austria; 4 Department of Cognition, Emotion, and Methods in Psychology, Faculty of Psychology, University of Vienna, 1010 Vienna, Austria; 5 Faculdad de Ciencias de la Salud, Universidad Europea de Valencia, 46010 Valencia, Spain; 6 Department of Psychology, University of Michigan, Ann Arbor, MI 48109 USA; 7 McGovern Institute for Brain Research, Massachusetts Institute of Technology, Cambridge, MA 02139 USA; 8 Department of Psychology, University of California Berkeley, Berkeley, CA 94704 USA; 9 Speech and Hearing Bioscience and Technology, Harvard University, Cambridge, MA USA; 10 School of Education, University of California, Davis, Davis, CA 95616 USA; 11 Brain Imaging Research Center, University of Connecticut, Storrs, CT 06269 USA; 12 Departments of Mathematics, Neuroscience, Psychiatry, Educational Psychology, Pediatrics, Computer Science and Engineering, University of Connecticut, Storrs, CT 06269 USA; 13 Haskins Laboratories, New Haven, CT 06511 USA

**Keywords:** Psychology, Human behaviour, Language

## Abstract

Consequences of multilingualism vary from offering cognitive benefits to poor educational and cognitive outcomes. One aspect of multilingualism that has not been systematically examined is the typology of multilinguals' languages: Do differences and similarities between languages multilinguals are exposed to contribute to the development of their cognition and brain? We investigated *n* = 162 5–6-year-olds with various language backgrounds on a monolingual-to-quintilingual continuum. Our results show that typological linguistic diversity can be related to expressive vocabulary knowledge in the dominant language. On neural level, it relates to brain activation patterns in (among others) the PGa area in the bilateral IPL, a brain region previously associated with multilingual experience, but never with language typology. We propose an ecologically valid way of describing the continuum of multilingual language experience and provide evidence for both the cognition and the brain of multilingual kindergartners to be related to the typological linguistic diversity of their environment.

## Introduction

Childhood multilingualism is fundamentally beneficial for communication and in some groups has been linked to cognitive benefits^1^. At the same time, many multilingual speakers experience inequality in language proficiency and educational attainment^2,3^. An issue so far overlooked by the majority of experimental studies, and database statistics alike, is the diversity of language exposure of the multilingual speakers: most studies operationalize bi-/multilingualism as a categorical variable^4,5^ and there is a notable dearth of evidence concerning the impact of **language typology** on multilingual (neuro)cognitive development. In the current study, we take the first step to systematically investigate the effects of linguistic diversity in multilinguals and take their lexical development as a test ground for the effects of typology of multilinguals' other languages on the functioning of the dominant language system.

The majority of information that proficient language users store relates to lexical semantics^6^. A multilingual’s accumulated lexical knowledge is distributed over more than one language. A common finding in the bilingual and multilingual literature is that individuals who know more than one language perform poorer than monolinguals on standardized vocabulary tests in one language due to differences in the proportion of exposure^7,8^. For example, Cattani et al.^7^ estimate a 60% L1 exposure threshold above which bilinguals perform like monolinguals in the L1: if—for example—an English–Spanish bilingual child is exposed to English and Spanish at a 70–30% ratio, their English vocabulary scores should fall within the range of a monolingual English child, while their Spanish score would be significantly lower than the Spanish monolingual norm. Other critical factors may include non-verbal intelligence and SES. Typological distance i.e., the relative similarities and differences between languages have received substantially less attention. Our focus is on whether overlaps between multilingual children’s languages contribute to the behavioral and neural signatures of lexical knowledge and processing in their dominant (L1) language (defined here as the language to which they were exposed from birth and the language of their environment). We aim to better and more comprehensively understand the contributions of multilinguals’ environment to language development, with particular attention to the combined effects of the amount of exposure to other languages and the lexical overlaps between them.

Compared to work with bilingual populations (agreeing that the smaller the distance, the greater the benefit to vocabulary in at least one language)^9–11^, direct evidence for the role of typological distance on L1 in *multilinguals* is virtually nonexistent. One study argued that multilingualism may in fact have positive effects on L1 as multilinguals acquire L1 vocabulary indirectly through L2, outweighing vocabulary loss through decreased L1 exposure^12^. This effect was especially salient for L2s with a relatively small lexical distance with respect to the L1 (i.e., those sharing many vocabulary items). However, the study reported effects of the “best foreign language”, not accounting for lexical overlaps between participants' other languages.

In terms of L1 lexical processing, multilinguals’ processing system is not selective: given a certain proficiency level, other languages can influence multilinguals’ L1 (dominant language) processing^13–16^. At the neural level, L1 processing among bilingual research participants is different from that of monolinguals (see^17^ for an overview). Further, overall higher levels of neural activity in bilinguals compared to monolinguals across a broad range of language and non-language regions have been tied to increased language processing demands in bilingual compared with monolingual participants, a phenomenon referred to as the brain’s “bilingual signature”^18^.

Whether and to what degree these effects apply to multilinguals with different language combinations is, to the best of our knowledge, unknown. Studies tackling typological distance effects at the neural level have concentrated on overlaps and dissociations in neural activity across L1 and L2, and not processing differences as a function of multilinguals’ experience. Although some studies have reported that processing languages with varied levels of similarity may converge on the same neuronal populations irrespective of linguistic distance^19^, others have tied larger linguistic distance to the use of additional neural resources during reading^20^, or syntactic processing^21^. Others yet provide evidence for stronger leftward lateralization for L2 auditory processing when L2 is more similar to L1^22^. Overall, the available research indirectly suggests that the more diverse one's language experience is, the more neural resources are necessary for language processing.

The current study seeks to fill the gap in our understanding of multilingual lexical processing. Specifically, we ask whether typology and relative lexical distance between *multiple* languages may explain variance in L1 vocabulary. Crucially, we combine measures of lexical distance with participants’ relative language exposure as operationalized by time. We aim to establish the behavioral and neural signatures of typological linguistic diversity of the environment in which multilingual children grow up and develop their language skills.

**Figure 1:**
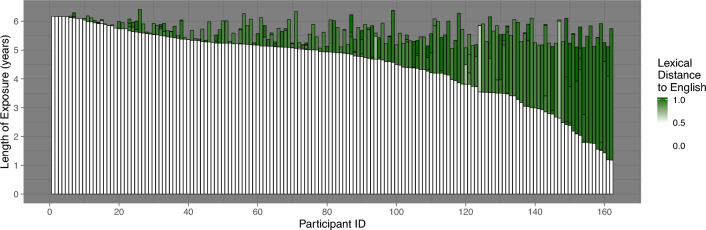
Illustration of the sample's language background. Each bar represents a single participant; the height of the whole bar represents their age; the height of the white part—their cumulative length of Exposure to English; Cumulative length of exposure to other languages is represented by the height of the green part within each bar; the shade of the green stands for the lexical distance of each language to English (indexed by LDND), the darker the shade, the more lexically distant from English the language. Prior to plotting, data was sorted by the Cumulative Length of Exposure to English values; consequently, data of children exposed to English the longest can be found on the left-hand side of the figure, the right-hand side includes data from participants with the least exposure to English.

Treating multilingualism as a spectrum of experiences and not a dichotomous category^23^, our approach is to describe multilingual children’s language experiences as precisely as possible. We capitalize on the vast linguistic diversity (in total 37 different languages) present in our sample of 5–6-year-old children (see Fig. 1) recruited from monolingual and multilingual community and school contexts, where participants had exposure to anywhere from one to five languages. We calculated the length of cumulative exposure to these languages and gathered information about relative typological distances between them based on the languages’ lexicons (see section on Independent measures for details). From these data, we derived three indices representing children’s cumulative language exposure: (i) proportion of cumulative length of exposure to English, (ii) diversity of language exposure, describing the variability of language input irrespective of language combinations but accounting for cumulative length of exposure to all languages, and (iii) typological diversity of language exposure, an index accounting for both the typological distance between participants’ languages *and* relative lengths of exposure. Using English receptive and expressive vocabulary scores as our outcome variables on one hand, and cortical activation levels during an English low-level, receptive lexical functional magnetic resonance imaging (fMRI) task on the other, we assessed whether the typological diversity of language exposure index would account for variance above and beyond the other two indices.

Our approach is novel in several respects. From a methodological point of view, in order to combine the different aspects of multilinguals’ environment, we leverage computational tools from information technology—Shannon’s^24^ entropy, and from the study of ecological diversity—Rao’s^25^ quadratic entropy index of diversity, thus offering a pioneering, multi-dimensional quantification of language exposure in multilinguals. By modelling language exposure of our entire sample, and not handpicking our groups of interest, we offer an ecologically valid way of describing the multilingual language experience, an approach rarely followed but highly called for in the multilingual literature. To the best of our knowledge, we are the first to investigate typological distance effects in multilingual children, accounting for relations between all their languages. We additionally supplement frequentist analyses with Bayesian approaches that allow us to discuss relative evidence for our effects of interest, or lack thereof. Furthermore, we offer insight not only into the behavioral manifestations of multilingualism but also its neural correlates, thus filling an important gap in research on L2-learning-induced neuroplasticity driven by language typology. Lastly, our methods have been pre-registered prior to data analysis (https://osf.io/7wq3k), safeguarding the transparency and reproducibility of our approach.

According to our pre-registered hypotheses, we expect to: (1) replicate the established correlations between children’s L1 (English) vocabulary knowledge and measures accounting for the relative proportion of time they were exposed to English. Secondly, (2) the measure of language exposure that accounts for relative length of exposure and linguistic distance will explain more variance in children’s vocabulary knowledge and lexical processing neuroimaging data than the measures modeling relative time only. Lastly, (3) linguistic distance will moderate the relationship between language exposure and behavioral and neural markers of lexical processing. Specifically, exposure to more distant languages will result in (a) lower English vocabulary scores than would be expected based on exposure data alone, and (b) L1 neural activation levels will be contingent on the degree of linguistic overlap between the languages (i.e., neural signature of bilingualism).

## Methods

### Data collection procedures

All procedures were approved by the University of California San Francisco Institutional Review Board. All procedures were carried out in accordance with guidelines and regulations of the University of California San Francisco Institutional Review Board. Informed consent was obtained from all subjects' legal guardians. Behavioral assessments were administered individually by trained research staff. Participants were compensated for their participation in the behavioral and neuroimaging sessions separately. Neuroimaging data were collected using a 3-T Siemens Prisma Fit MRI scanner with a 64-channel head coil. Children were familiarized with the experimental procedures prior to scanning. We used padding to dampen scanner noise and reduce motion and delivered sound through an MRI compatible sound-system. Participants' responses were recorded with a button box. The task was created and presented in E-Prime software. Whole-brain functional images were acquired using a gradient-echo echo-planar pulse sequence (TR = 1250 ms, TE = 33.40 ms, FA = 45°, FOV = 220 mm, voxel size = 2.2 mm^3^, 64 contiguous 2.20 mm axial slices, 0 mm inter-slice gap). High resolution T1-weighted anatomical images were acquired with a matrix size of 256*256; 160 contiguous axial slices; voxel resolution 1 mm, TR = 2300 ms, TE = 2.98 ms, T1 = 900 ms; and FA = 9.

### Participants

184 children participated in at least one behavioral assessment and were invited for neuroimaging; the available behavioral dataset (including all dependent and independent variables) consisted of *N* = 167 data points. Children's L1 status was determined based on age of acquisition. Therefore to keep the L1 constant across the whole sample, we removed data of 5 participants who were not exposed to English from birth. The resulting final sample included *N* = 162 participants (65 females) in ages 5.05–6.41 years (*M* = 5.68). Children's socio-economic status (SES) was indexed as the average of the highest level of education in years attained by each parent (in 4 cases, data was available for only one of the parents; SES in these cases was indexed by the highest level of education in years attained by the parent who provided the data). The participants came from a high socio-economic background, their parents having completed *Mdn* = 17; *SD* = 1.99 years of education. The children were exposed to *N* = 37 different languages at various lengths, see Fig. 1; Table 1 presents the breakdown of participants by number of languages to which they were exposed; lexical similarity between all languages is presented in Fig. S1. 157 children took part in neuroimaging data collection. Due to movement during the fMRI, failure to complete the task, hardware changes, lower than chance task accuracy and missing data, 92 datasets were available for higher-level analyses.

**Table 1:** Breakdown of participants by number of languages to which they were exposed according to the administered parental language exposure questionnaire.

Number of languages	1	2	3	4	5
Number of participants	11	79	47	18	7

### Independent measures

Language exposure data were derived from the ‘Amount of language exposure in the past’ section of the Bilingual Language Experience Calculator (BiLEC)^26^, see Supplementary Materials for details. Lexical distance between languages was collected through the freely available ASJP Database^27^. ASJP provides an objective, data-driven evaluation of similarity between words with the same meaning from different languages by comparing lists of words relating to 40 concepts specified in^28^. The word lists are transcribed into a simplified standard orthography^29^ and compared with each other based on their Levenshtein Distance^30^ (i.e., minimum number of insertions, deletions or substitutions necessary to convert one word into another). Levenshtein Distance Normalized Divided (LDND) was used in the present study, given its robustness against chance resemblances^31^. LDND corrects for differences in word length by dividing the raw Levenshtein distance by the length of the longer of the two words (resulting in LDN—Levenstein Distance Normalized), and divides it by the average LDN of words not referring to the same concept. The distances were computed using the program ASJP.R by Wichmann (https://github.com/Sokiwi/InteractiveASJP01) and are presented in Fig. S1; Table S1 lists LDND between English and all languages to which the participants were exposed.

#### Indices

(i) Cumulative length of exposure to English was calculated using information gathered with the BiLEC form (see above). Details on exact calculations can be found in the instrument’s manual available online. Per authors’ instructions, country-specific and participant-specific data were changed in the form, including values for average number of hours at school, and number of weeks’ holiday per year. In our analyses, we used the *proportion* of Cumulative Length of Exposure to English, calculated by dividing the total English exposure BiLEC-derived value (in years) by participant’s age at testing. (ii) Diversity of language exposure indexed variability of each participant’s language exposure, irrespective of language combinations, but accounting for cumulative length of exposure to all languages. We computed it using the Shannon’s entropy (*H*) equation^24^:

$$H=-{\sum }_{\mathrm{i}=1}^{\mathrm{n}}{p}_{i}\,{ log}_{2}\,{p}_{i}$$

where, *n* stands for the total number of languages a participant has been exposed to and *p*_*i*_ is the proportion of time (in years) a participant was exposed to that language during their life. We calculated the "Diversity" index using the entropy R package^32^. (iii) Typological Diversity of language exposure was created using Rao's quadratic entropy equation (*QE*)^25^. The summed lexical distances between all language pairs for each child were weighted by the BiLEC-derived length of exposure to each language as follows: If the proportion of cumulative length of exposure to *i*-th language in a child’s repertoire is *p*_*i*_ and the dissimilarity between language *i* and *j* is *d*_*ij*_, then "Typological Diversity" has the form:

$$QE = {\sum }_{i=1}^{S} {\sum }_{j=1}^{S} {d}_{ij }{p}_{i }{p}_{j}$$

where *d*_*ij*_ varies from 0 (i.e., the two languages have exactly the same lexicons) to 1 (i.e., where the two languages have completely different lexicons). We calculated the "Typological Diversity" index using the SYNCSA R package^33^.

### Dependent measures

All collected data were part of a larger test battery examining children’s language and cognitive development. Receptive (Standard American) English vocabulary was measured with the Peabody Picture Vocabulary Test (PPVT), 4th Edition, Form A^34^. Expressive English vocabulary was measured with the Picture Vocabulary subtest of the Woodcock-Johnson, 4th Edition (WJ-IV-PV), Tests of Oral Language^35^. Outliers for the PPVT and WJ-IV-PV scores were defined as ± 3 SD from the mean and one data-point was removed from the analysis of the WJ-IV-PV. The scores on both tasks were correlated with each other at *r* = 0.77, *p* < 0.001. For a full description of fMRI experimental task, the English Word Match Task see^36^ and Supplementary Materials.

### fMRI pre-processing

Imaging data were processed using FSL^37^. Pre-processing using FEAT included: removal of the first 11 volumes for signal equilibration (173 volumes retained); motion correction using MCFLIRT^38^; non-brain removal using BET^39^; grand-mean intensity normalization of the entire 4D dataset by a single multiplicative factor; spatial smoothing (Gaussian kernel of 5 mm FWHM); B0 unwarping using BBR^38,40^ (performing simultaneous registration to the high resolution T1-image; rigid body, 6 degrees of freedom). T1-images were registered to an age-appropriate pediatric template (MNI NIHPD asymmetrical, 4.5–8.5 years^41^) using 12-parameter affine transformation and non-linear registration with a warp resolution of 10 mm in FNIRT^42,43^. ICA-AROMA^44^ identified and removed head motion-related artefacts from fMRI data that passed motion quality control (absolute mean displacement < 5 mm). The de-noised data were then high-pass filtered with a cut-off of 36 s (0.036 Hz) and the registration parameters were reapplied.

### Data analysis

#### fMRI Single-level statistical analysis

Time-series statistical analysis was carried out using FILM with local autocorrelation correction^45^. The design matrices for each run for each participant included task blocks as events of interest with signal from white matter and cerebrospinal fluid as nuisance regressors. Relative levels of BOLD signal during task blocks compared to rest blocks derived from the single-level statistical analyses of the experimental task were used as dependent variables in the group-level analyses.

#### Covariates of no-interest

Participant’s age at the time of testing, their gender, SES and nonverbal reasoning skills measured by the Kauffman Brief Intelligence Test: Matrices, 2nd Edition (KBIT-2)^46^ were used as covariates of no-interest in all models, see Table S3. For the group-level neuroimaging data analysis, we additionally included participants’ handedness, d’ scores calculated from their responses on the fMRI task and headphones model used during data acquisition.

#### Inference criteria

Three types of inference criteria were used to confirm or reject our hypotheses: frequentist, Bayesian and visual inspection of the data. For frequentist approaches, we used the standard *p* < 0.05 criterion for determining if the models suggest that the results are significantly different from those expected if the null hypothesis were correct. Bayes factors (BFs) were used and interpreted following^47–52^ and informed our models selection. We compared models with the Bayesian information criterion (BIC)^53^ and the difference between two BICs was converted into a Bayes factor using the equation below, following^47^:

$${BF}_{10}=\mathrm{exp}\frac{\Delta {BIC}_{01}}{2}$$

Neuroimaging data were assessed using log Bayes factor (LBF) voxel-wise maps. Thresholds for LBFs of 0, 1, 3 or 5 correspond to BFs of around 1, 3, 20 or 150 and evidence in favor of model *m*_*1*_ against model *m*_*0*_ cf. ^54^ and can be labeled as "anecdotal", "positive", "strong" and "very strong" respectively. We compared the models quantitively by looking at the number of voxels displaying model preferences; all voxel-wise visualizations of the BOLD data were further subject to visual interpretation (in conjunction with the use of anatomical atlases available via the visualization software used).

#### Hypothesis 1

**Figure 2:**
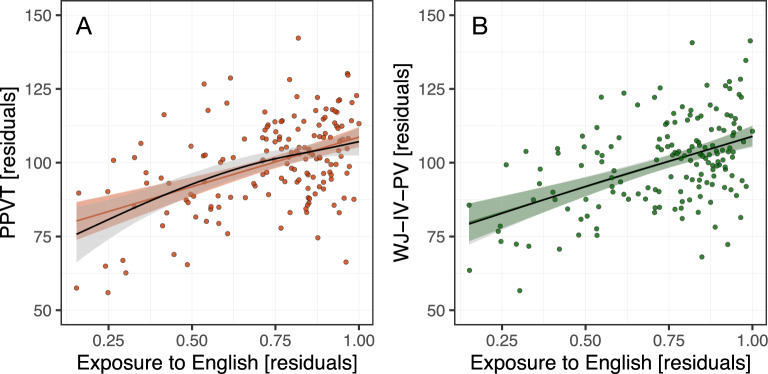
Relationship between the proportion of Cumulative Length of Exposure to English and (**A**) receptive (PPVT) and (**B**) expressive vocabulary scores (WJ-IV-PV), both controlled for age, gender, SES and non-verbal intelligence, standardized and centered around 100 with *SD* = 15 for visualizations. Next to individual participants' data, we present the linear (colored lines) and smooth (black lines) terms for Cumulative Length of Exposure to English. NB., the smooth term for Exposure to English in the WJ-IV-PV model in (**B**), overlaps almost fully with the linear fit. See Table S5 for details on the model comparison procedure.

Linear models with covariates of no-interest were used to determine the effect of cumulative length of exposure to English on L1 vocabulary: (i) receptive vocabulary scores (indexed by PPVT) and (ii) expressive vocabulary scores (indexed by WJ-IV-PV). Our model comparison procedure^47^ involved (in each case) fitting a baseline model (containing covariates of no-interest) and a model additionally containing the "Exposure to English" variable. Exposure to English was highly predictive of both the receptive and expressive vocabulary scores, see Fig. 2 and Table S5. Details on the cluster analysis, curve-fitting analyses and breakpoint discovery procedure are presented in Supplementary Materials.

#### Hypothesis 2

We hypothesized that more variance in children’s vocabulary knowledge and lexical processing would be explained by accounting for the typological distance between their languages than by modelling the relative exposure time only. Table S2 summarizes correlation coefficients between the three language exposure indices. Of note, the indices are all based on information on the length(s) of language exposure and are thus highly correlated. Therefore, we expected the differences in the amount of explained variance between the models of interest to be modest. After inspecting the calculated language exposure indices, we excluded participants who were exposed to English less than 50% of their lives (*n* = 18 for the behavioral data, and *n* = 10 for fMRI), because the "Diversity" and "Typological Diversity" indices would yield the same values (e.g., for two Cantonese—English bilingual individuals exposed to the two languages at the relative ratios of 0.4–0.6 and 0.6–0.4). The behavioral results reported above were obtained from *n* = 144 participants for PPVT, and *n* = 142 for WJ-IV-PV (one missing value, and one outlier was removed), and the fMRI results from *n* = 82 participants, whose data survived quality control and who were exposed to English more than 50% of their lives. We conducted separate analyses for each of the two outcome variables (PPVT and WJ-IV-PV). For each, four linear models were specified and estimated using R:

(*H2*-*m*_*0*_) baseline model (including covariates of no-interest only),

(*H2*-*m*_*1*_) baseline + Exposure to English,

(*H2-m*_*2*_) baseline + Diversity,

(*H2-m*_*3*_) baseline + Typological Diversity.﻿

Kolmogorov-Smirnov normality tests were run on all models' residuals, revealing that parametric linear models were appropriate for the present data (all *p*s > 0.6).

Using an English Word Match Task, we aimed at establishing which of our three indices of language exposure was the best at predicting group-level BOLD data. Four General Linear Models (GLMs) were specified and estimated with Statistical Parametric Mapping software (SPM12; Wellcome Centre for Human Neuroimaging, London, UK; https://www.fil.ion.ucl.ac.uk/spm/), following the makeup of the linear models in the behavioral data analysis (with additional, task-related covariates of no-interest). Subsequently, we followed model assessment procedures described in ^54^ and implemented in the MACS toolbox in SPM. For each model we first calculated voxel-wise cross-validated log model evidence (cvLME) based on a partition of the data described by each GLM. For group-level GLMs, the design matrix was split in two parts using random splitting (with the constraint that both parts have the same size). MATLAB’s random number generator was seeded with the number of datasets to ensure the same splitting for different models describing the same data. After generating the cvLME maps, we compared the models using log Bayes factor (LBF) maps by subtracting two cvLME maps from each other (with fslmaths). The models were compared iteratively, taking the complexity of our indices into account. We looked for voxels preferring: *H2-m*_*1*_ over *H2-m*_*0*_ (and vice versa); *H2-m*_*2*_ over *H2-m*_*1*_ and over *H2-m*_*0*_ (and vice versa); *H2-m*_*3*_ over *H2-m*_*2*_, over *H2-m*_*1*_ and over *H2-m*_*0*_ (and vice versa). Since Hypothesis 2 stated that more variance in children’s lexical processing neuroimaging data would be explained by the measure of language exposure that accounts for the typological distance between their languages (as in *H2-m*_*3*_) than by measures modelling the time aspect only (as in *H2-m*_*1*_ and *H2-m*_*2*_), the comparisons crucial for answering our research questions were ones involving *H2-m*_*3*_ against *H2-m*_*2*_, and *H2-m*_*1.*_ The LBF thresholds were first set to LBF > 3 (i.e., providing "strong evidence" in favor of a model cf. ^54^). Given the very strong correlations between our three indices of interest (Table S2), if a comparison resulted in no surviving voxels, they were lowered to LBF > 1.5. Cluster formation was performed with FSL's cluster tool, region-of-interest (ROI) analyses were performed with FSL’s featquery.

#### Hypothesis 3

Our last hypothesis stated that linguistic distance would moderate the relationship between language exposure and behavioral and neural markers of lexical processing. Given that being exposed to more than 2 languages means that these languages’ lexicons also ‘interact’ with one another, we tested this hypothesis in a sub-sample of our participants (*n* = 127 for PPVT, *n* = 126 for WJ-IV-PV, and *n* = 73 for fMRI data), selected based on the language exposure data with the criterion that they had been exposed to only two languages for at least 95% of their lives. The distance of the different L2s in our database to English was collected through the ASJP database^27^ and represented by LDND^31^. For each outcome variable, four linear models were specified and estimated using R:

(*H3-m*_*0*_) baseline model (including covariates of no-interest only, see Methods),

(*H3-m*_*1*_) baseline + Exposure to English,

(*H3-m*_*2*_) baseline + Exposure to English + L2 Distance to English,

(*H3-m*_*3*_) baseline + Exposure to English + L2 Distance to English + [Exposure to English x L2 Distance to English] (henceforth "Interaction model").

Kolmogorov-Smirnov normality tests run on models' residuals confirmed that parametric linear models were appropriate for the data (all *p*s > 0.7).

## Results

### Hypothesis 1

We replicated the established correlations between children’s L1 (English) receptive and expressive vocabulary knowledge (measured by PPVT and WJ-IV-PV respectively) and the relative proportion of time they were exposed to English, see Fig. 2. The BFs offered "overwhelming" evidence for this association in both cases. To replicate the finding of a 60% exposure to English cut-off point above which bilinguals (multilinguals in our case) should perform on a par with English monolinguals^7,8^, we followed: a two-stage cluster analysis (reproducing the methods from^7^); curve-fitting analyses; and a breakpoint discovery procedure (see Supplementary Materials for details). The results suggest that the relationship between cumulative length of Exposure to English, and English receptive and expressive vocabulary scores in our sample is linear and there seem to be no threshold of minimal exposure length above which the vocabulary scores would not increase further.

### Hypothesis 2

*Behavioral data* We conducted separate analyses for each of the two outcome variables (PPVT and WJ-IV-PV). Four linear models were specified and estimated using R (see Methods).

According to a frequentist analysis of the data, all eight overall regression models were statistically significant (all *p*s < 0.01); so were the main effects of all three language exposure indices, in both performed analyses, see Table 2. Effect sizes (Cohen’s *f*^*2*^) were consistently "small"; for the Receptive Vocabulary models (*f*^2^ = 0.100, *f*^2^ = 0.060, and *f*^2^ = 0.083 for Exposure to English, Diversity and Typological Diversity respectively); and for Expressive Vocabulary (*f*^2^ = 0.065, *f*^2^ = 0.041, and *f*^2^ = 0.063 for Exposure to English, Diversity and Typological Diversity, respectively). Since based on the frequentist analysis we were not able to unequivocally reject or confirm our hypothesis, the fitted models were compared with each other incrementally. The model comparison procedure showed that receptive vocabulary was by far best predicted by the simplest of our language exposure indices, Exposure to English. In contrast, expressive L1 vocabulary could be best predicted by language exposure index incorporating information about linguistic typology of languages a child has been exposed to. The Typological Diversity model (*H2-m*_*3*_) provided "very strong"^55^ evidence against the baseline model (*H2-m*_*0*_), and direct comparison between the Typological Diversity (*H2-m*_*3*_) and Exposure to English (*H2-m*_*1*_) models revealed that *H2-m*_*3*_ slightly outperformed *H2-m*_*1*_.

**Table 2:** Multiple regression model parameters for the receptive and expressive vocabulary scores, as predicted by the three language exposure indices (Exposure to English, Diversity and Typological Diversity), and model comparison results (additional variance explained and *BF*_*10*_ values).

Models	β	*t*	*DF*	*p*	*ΔR*^*2*^	*BF*_*10*_
**Receptive vocabulary ~ language exposure indices**						
(*H2-m*_*1*_) Exposure to English	35.554	3.722	5,138	< .001		
*versus* (*H2-m*_*0*_) Baseline					0.065	81.722
(*H2-m*_*2*_) Diversity	− 15.32	− 2.885	5,138	= .005		
*versus* (*H2*-*m*_*0*_) Baseline					0.039	5.651
*versus* (*H2*-*m*_*1*_) Exposure to English					− 0.027	0.069
(*H2-m*_*3*_) Typological Diversity	− 29.882	− 3.385	5,138	< .001		
*versus* (*H2-m*_*0*_) Baseline					0.054	25.985
*versus* (*H2-m*_*1*_) Exposure to English					− 0.011	0.317
*versus* (*H2-m*_*2*_) Diversity					0.015	4.598
**Expressive vocabulary ~ language exposure indices**						
(*H2-m*_*1*_) Exposure to English	6.333	3.571	5,136	< .001		
versus (*H2-m*_*0*_) Baseline					0.072	48.612
(*H2-m*_*2*_) Diversity	− 2.994	− 3.042	5,136	= .003		
*versus* (*H2-m*_*0*_) Baseline					0.052	8.993
*versus* (*H2-m*_*1*_) Exposure to English					− 0.020	0.185
(*H2-m*_*3*_) Typological Diversity	− 5.841	− 3.582	5,136	< .001		
*versus* (*H2-m*_*0*_) Baseline					0.073	50.568
*versus* (*H2-m*_*1*_) Exposure to English					< 0.001	1.040
*versus* (*H2*-*m*_*2*_) Diversity					0.021	5.622

*fMRI data* Four models (as in the behavioral data analysis above, plus fMRI-task-related covariates of no-interest, see Methods) were specified on fMRI data collected during an English Word Match Task.

**Figure 3:**
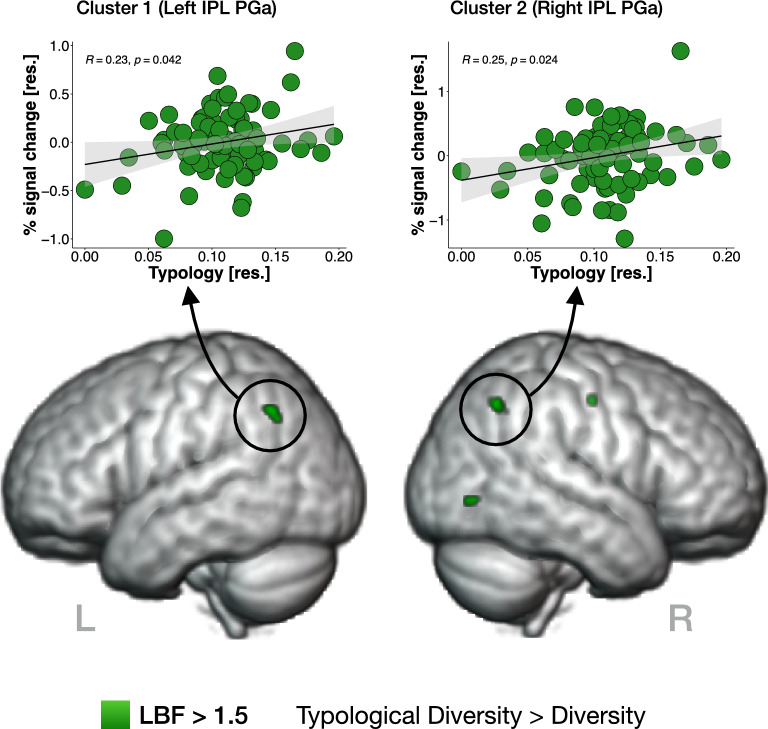
Effect of language typology: presented on the bottom are clusters of voxels (in green) showing preference for the model including typological information on participants' language background ("Typological Diversity") over a model accounting for cumulative length of exposure to all languages ("Diversity"). Graphs on top show the percentage signal change values for the BOLD signal recorded during the English Word Match Task in bilateral IPL PGa areas, as a function of residual values of Typology (i.e., Typological Diversity index controlled for covariates of no-interest and length of exposure to all languages). NB. the percentage signal change values in the two remaining clusters are presented in the Supplementary Materials (see Fig. S4).

With respect to the number of voxels displaying model preferences, all three language exposure indices explained more variance than the baseline model. The highest number of voxels preferred the "Exposure to English" model (*H2-m*_*1*_), followed by models with "Typological Diversity" (*H2-m*_*3*_) and "Diversity" (*H2-m*_*2*_), see Table 3. The comparison between *H2-m*_*3*_ against *H2-m*_*2*_ showed three times more voxels than the comparison of *H2-m*_*2*_ against *H2-m*_*3*_, and about 50 more for *H2-m*_*3*_ against *H2-m*_*1*_ than *H2-m*_*1*_ against *H2-m*_*3*_, thus confirming Hypothesis 2. To elaborate on the topography of these effects, we report clusters of voxels surviving the higher threshold and consisting of at least 10 voxels, see Table 4 and Fig. 3.

**Table 3:** Number of voxels showing model preference according to the specified LBF threshold for each of the performed model comparisons testing Hypothesis 2.

	*H2-m*_*0*_ >		*H2-m*_*1*_ >		*H2-m*_*2*_ >		*H2-m*_*3*_ >	
	LBF > 3	LBF > 1.5	LBF > 3	LBF > 1.5	LBF > 3	LBF > 1.5	LBF > 3	LBF > 1.5
*H2-m*_*0*_	-	-	302	2242	225	1769	239	1910
*H2-m*_*1*_	7798	39,560	-	-	131	3421	0	375
*H2-m*_*2*_	7403	35,715	44	2317	-	-	0	168
*H2-m*_*3*_	6925	36,220	2	116	0	116	-	-

The information included in the "Typological Diversity" language exposure index was reflected in the activation patterns of several brain regions during the English Word Match Task. Notably, typological relations between multilinguals' languages seemed to modulate the activity of the PGa area in the Inferior Parietal Lobule (IPL) bilaterally and of two small clusters in the right visual and premotor cortices, as reflected in LBF maps generated by comparing *H2-m*_*3*_ over *H2-m*_*2*_*.* Follow-up region-of-interest (ROI) analyses (Fig. 3, top panel) revealed that the percentage signal change values for the BOLD signal recorded during the English Word Match Task in bilateral IPL PGa areas was linearly and positively related to the amount of typological information on participants' languages. This was established by performing partial correlation analyses (controlling for covariates of no-interest) between the percentage BOLD signal change values in each ROI and residual values of Typology (i.e., regressing out the length of exposure to all languages, as quantified by the "Diversity" index, from the "Typological Diversity" index). In other words, the more typologically diverse our participants' language background was, the more BOLD signal was observed during L1 (English) lexical processing. A broader network of areas showed preference for the typological information than for English exposure information alone, see Table 4 and Fig. S5.

**Table 4:** Brain areas showing model preferences according to the specified LBF thresholds (and with clusters consisting of at least 10 voxels, ordered by size). The x, y and z coordinates are in the age-appropriate pediatric MNI NIHPD space^41^, the regions were labelled according to Harvard–Oxford Cortical and Subcortical Structural Atlases, Jülich Histological Atlas and Cerebellar Atlas (all implemented within FSLeyes, part of FSL, after non-linearly transforming the atlases to the pediatric space).

Cortical region	L/R	size (voxels)	LBF_max_	Peak location (voxels)		
				x	y	z
**(*H2-m***_***3***_**) Typological Diversity > (*H2-m***_***2***_**) Diversity**						
LBF > 3						
**–**	**–**	**–**	**–**	**–**	**–**	**–**
LBF > 1.5						
1. Inferior parietal lobule PGa	L	28	2.11	69	33	57
2. Inferior parietal lobule PGa	R	22	2.21	17	32	59
3. Visual cortex V5	R	15	1.97	25	26	35
4. Premotor cortex BA6	R	10	2.19	40	57	60
** (*H2-m***_***3***_**) Typological Diversity > (*H2-m***_***1***_**) Exposure to English**						
LBF > 3						
**–**	**–**	**–**	**–**	**–**	**–**	**–**
LBF > 1.5						
1. Superior parietal lobule 5 M	L	56	2.78	53	40	68
2. Visual cortex V5	R	29	1.89	18	31	42
3. Lingual gyrus	L	24	2.58	54	40	36
4. Cerebellum, VIIIa	L	18	2.41	54	32	7
5. Inferior parietal lobule PF	R	17	2.61	11	44	53
6. Inferior parietal lobule PFt	L	17	1.97	71	47	59
7. Cerebellum, VIIIb	R	16	2.75	34	39	6
8. Visual cortex V1 BA17	R	16	1.86	36	25	40
9. Superior parietal lobule 5 Ci	R	13	2.22	37	40	64
10 Visual cortex V1 BA17	R	11	1.76	36	32	41
11. Subcallosal cortex	R	11	2.15	41	74	30
12. Hippocampus subiculum	R	10	1.71	31	52	25
**(*H2-m***_***2***_**) Diversity > (*H2-m***_***3***_**) Typological Diversity**						
LBF > 3						
**–**	**–**	**–**	**–**	**–**	**–**	**–**
LBF > 1.5						
1. Hippocampus (cornu ammonis)	R	33	2.73	29	53	26
2. Lingual gyrus	L	11	2.09	54	40	35
**(*H2-m***_***1***_**) Exposure to English > (*H2-m***_***3***_**) Typological Diversity**						
LBF > 3						
**–**	**–**	**–**	**–**	**–**	**–**	**–**
LBF > 1.5						
1. Premotor cortex BA6	R	30	3.21	40	57	60
2. Temporal pole	R	12	1.97	21	71	23

### Hypothesis 3

*Behavioral data* We tested the moderating effect of language distance on the relationship between the length of language exposure and vocabulary scores in a bilingual sub-sample of participants, by looking for significant interactions between the L2 Distance variable and the length of Exposure to English variable using multiple regression analyses. We also ran models including main effects of both variables only. For each outcome variable, four linear models were specified and estimated using R (see Methods). According to the frequentist analysis, all eight overall regression models were statistically significant (*p*s < 0.001, Table 5). For PPVT scores, for Exposure to English and L2 Distance, respectively, effect sizes were equal to *f*^2^ = 0.17, and *f*^2^ = 0.03; and *f*^2^ = 0.20, *f*^2^ = 0.04, for WJ-IV-PV. This represents a medium effect for Exposure to English and a small effect for L2 Distance. For both the PPVT and WJ-IV-PV scores, the models including Exposure to English (*H3-m*_*1*_), and Exposure to English and L2 Distance to English (*H3-m*_*2*_) offered "overwhelmingly" more evidence than the baseline only model (*H3-m*_*0*_). The models including the interaction term (*H3-m*_*3*_) were also far better than the baseline models (*H3-m*_*0*_) but had worse fits than models testing for main effect of L2 distance and English exposure only (*H3-m*_*2*_). Their *BFs* showed that the data provided substantially more evidence for the relationship between the length of language exposure and PPVT and WJ-IV-PV scores not to be moderated by the L2 distance variable, thus rejecting Hypothesis 3. English receptive vocabulary was, again, best predicted by the model with Exposure to English index only: the model additionally including the L2 Distance variable (*H3-m*_*2*_) offered "moderately" less evidence than the Exposure to English model (*H3-m*_*1*_). However, for English expressive vocabulary scores, a model including main effect of L2 Distance to English and Exposure to English (*H3-m*_*2*_) did outperform the model with the length of Exposure to English variable only (*H3-m*_*1*_). In frequentist terms, L2 Distance to English did significantly predict expressive vocabulary scores *above and beyond* the English length of exposure variable. Notably, the effect of language typology was observed for the expressive but not receptive vocabulary scores, similarly to the analysis testing Hypothesis 2. Figure S6 shows the relationship between the receptive and expressive vocabulary scores and L2 Distance to English. The fact that the results point to a lack of an interaction between L2 Distance and L1 Length of Exposure, but L2 Distance is significantly related to the expressive vocabulary scores above and beyond the effect of L1 Length of Exposure provides an analogous replication of the results obtained from testing Hypothesis 2 and shows that typology is an independent predictor of L1 expressive vocabulary.

**Table 5:** Multiple regression models' parameters and *BF*_*10*_ testing Hypothesis 3 in a bilingual sub-sample of participants.

Models	*R* ^*2*^	*F*	*DF*	*t*	β	*p*	*ΔR* ^*2*^	*BF*_*10*_
**Receptive vocabulary (PPVT) ~ **								
(*H3-m*_*0*_) Baseline	0.236	10.74	4,122	–	–	–		
(*H3-m*_*1*_) Exposure to English	0.337	13.82	5,121	4.426	34.285	< .001		
*versus* (*H3-m*_*0*_) Baseline							0.101	1222.023
(*H3-m*_*2*_) Exposure to English + L2 distance to English	0.351	12.36	6,120	− 1.892	− 45.951	= .06		
*versus* (*H3-m*_*0*_) Baseline							0.115	700.736
*versus* (*H3-m*_*1*_) Exposure to English							0.014	0.573
(*H3-m*_*3*_) Exposure to English x L2 distance to English	0.346	10.54	7,119	0.407	49.103	= .68		
*versus* (*H3-m*_*0*_) Baseline							0.110	67.915
*versus* (*H3-m*_*1*_) Exposure to English							0.009	0.055
*versus* (*H3-m*_*2*_) Exposure to English + L2 Distance							− 0.005	0.097
**Expressive Vocabulary (WJ-IV-PV) ~ **								
(*H3-m*_*0*_) Baseline	0.064	3.13	4,121	–	–	–		
(*H3-m*_*1*_) Exposure to English	0.211	6.667	5,120	4.849	7.238	< .001		
versus (*H3-m*_*0*_) Baseline							0.147	6995.353
(*H3-m*_*2*_) Exposure to English + L2 distance to English	0.237	7.49	6,119	− 2.286	− 10.703	= .024		
versus (*H3-m*_*0*_) Baseline							0.174	9345.031
versus (*H3-m*_*1*_) Exposure to English							0.027	1.336
(*H3-m*_3_) Exposure to English x L2 distance to English	0.233	6.41	7,118	0.499	11.535	= .619		
versus (*H3-m*_*0*_) Baseline							0.169	950.760
versus (*H3-m*_*1*_) Exposure to English							0.022	0.136
versus (*H3-m*_*2*_) Exposure to English + L2 Distance							− 0.005	0.102

*fMRI data* For the English Word Match task performed during fMRI, four GLMs were specified and estimated in SPM12, following the linear models in the behavioral data analysis above (plus fMRI-task-related covariates of no-interest, see Methods). We followed the same analytical steps as for Hypothesis 2 analysis. All comparisons yielded results at the LBF > 3 threshold, providing "strong evidence" for brain areas being modulated by the specific combination of predictors included in each model, see Table S6. Crucially for the research question of the current study, both models including the L2 Distance variable, (*H3-m*_*2*_) and (*H3-m*_*3*_) could be tied to specific brain activity patterns during the English Word Match Task.

In support of Hypothesis 3, a set of voxels preferred the Interaction model (*H3-m*_*3*_) over the model with main effects of Exposure to English and L2 Distance only (*H3-m*_*2*_) at LBF > 3, see Fig S7, panel B and Table S7. The comparison between the model specifying the length of Exposure to English (*H3-m*_*1*_), and the model additionally including the L2 Distance variable (*H3-m*_*2*_), resulted in a broad network of areas showing preference for the typological information, see Fig. S7 and Table S7. Notably, the largest of the identified clusters was localized in the same region as the typology effect reported for Hypothesis 2 above. ROI analyses were further performed in the five largest of the identified clusters. Overall, the percentage BOLD signal change values were highest for participants with an L2 lexically closest to English; participants with an L2 most dissimilar from English showed highest deactivations in these ROIs, see Fig. S7, panel A.

## Discussion

Per Hypothesis 1, we replicated the established correlations between children’s L1 vocabulary knowledge and measures accounting for the relative proportion of L1 exposure. However, contrary to previous results reporting a 60% exposure threshold beyond which bilinguals perform like monolinguals in their L1^7,8^, our data suggest that the relationship between L1 vocabulary knowledge and L1 cumulative exposure is fully linear. We argue that our curve-fitting and breakpoint discovery procedure analyses accompanied by Bayesian inference provide sufficient evidence even for a lack of this effect. Notably however, a distinction needs to be made between "performance within monolingual range" (as proposed by^7,8^) and a linear relationship between the investigated variables. Our data suggest that even "functionally monolingual" children (i.e., those with very little exposure to L2) might increase their vocabulary knowledge as a function of longer exposure to a language. This finding may prove particularly relevant for multilingual populations whose vocabulary knowledge lags behind their peers due to SES, cognitive, or other extralinguistic factors.

Our data show that cumulative length of Exposure to English is the best predictor of receptive vocabulary knowledge, more so than an index encompassing multilinguals' experience with other languages. Expressive vocabulary, however, could be better predicted by a measure incorporating typological information. For a bilingual sub-sample of participants, the distance of their L2 to English predicted their scores *above and beyond* their cumulative length of Exposure to English.

Differential effects of L1-L2 typological distance on expressive *versus* receptive vocabulary have been reported previously^11^. A language distance index similar to ours (measuring overlap in word forms) was positively related to expressive but not receptive vocabulary scores (receptive vocabulary scores were tied to languages' morphological or syntactic structure). These effects were, however, found for transfer from the dominant (L1) to the non-dominant (L2) language, while we report L2-(and L3, L4 etc.)-towards-L1 effects. One explanation for this discrepancy might be participants' age (~ 2 years *versus* ~ 5–6 years); perhaps by the time children are 5 years old, their experience with additional languages might start to influence their L1 as well. The differential effects of typological information for expressive *versus* receptive vocabulary might also be tied to greater cognitive load for expressive as opposed to receptive language tasks and the fact that expressive vocabulary tasks rely more heavily on lexical access processes than receptive ones.

Our data show that sub-processes involved in word production might be aided by lexical similarity of multilinguals' other languages to the target language (or interfered with when a multilingual's languages are dissimilar from L1). Previous research has also associated receptive vocabulary knowledge with L2's distance to the target language^10^. Blom and colleagues used, however, a global language distance measure (categorizing L2s into "close" and "distant" to L1). It is plausible that a better representation of "close" languages in our sample might have resulted in stronger ties to receptive vocabulary knowledge (most languages present here would be categorized as "distant" by Blom et al.'s criteria; see also Supplementary Materials for an exploratory analysis on Spanish–English and Cantonese-English bilinguals). The facilitatory effect of small L2 distance for expressive vocabulary (and lack thereof for receptive vocabulary) reported here might inform future investigations of the so-called "receptive-expressive gap" in bilinguals' L1^56^.

Finally, our results bring nuance to the claim that multilingualism positively affects L1 vocabulary development^12^. Multilinguals may indeed acquire L1 vocabulary indirectly through their other languages if the languages share enough lexical items with L1, but our data do not support the claim that this indirect lexical acquisition outweighs vocabulary loss through decreased L1 exposure. If that were the case, we would have found evidence for an interaction between both variables, or a larger effect size of L2 Distance than of Exposure to English. We found that *f*^2^ value of L2 Distance was small while the effect of Exposure to English was of medium size, indicating that while small linguistic distance might to some degree help in vocabulary acquisition, multilingualism as a whole will not outweigh vocabulary loss through decreased L1 exposure.

Our fMRI analyses indicate the caudal part of IPL (area PGa) is sensitive to typological information describing participants' language experience. This effect was found bilaterally for the parametric effect of typological diversity on a monolingual-to-quintilingual continuum, and in the right hemisphere in the analysis focusing on bilinguals. According to Ben Shalom and Poeppel's^57^ tripartition for the involvement of the IPL in language tasks, its rostral part processes sounds and single phonemes, the middle part underlies morphological operations, and the caudal IPL areas determine the semantic content of words or sentences. This subdivision closely aligns with connectivity data^58^, cytoarchitectonic studies^59^, and transmitter receptor-based data^60^. In previous research on multilingualism and language learning, IPL has indeed been related to semantic and phonological processing^61^, decoding the meaning of words^62^, learning novel speech sounds^63^, and to support learning of grammar rules^64^. In the context of the Dual Stream Model of speech processing, IPL is part of the dorsal stream underlying the auditory–motor interface. Vocabulary acquisition may involve the IPL in "generating a sensory representation of the new word that codes the sequence of segments or syllables"^65^.

The caudal IPL, and especially the PGa area, has been repeatedly associated with language and multilingual experience, but never with typological linguistic diversity. Already in 1925, it was referred to as the "language talent area" by an Austrian neurologist Otto Pötzl, who associated it with the exceptionality of multilinguals^66^. In one of the first ever in-vivo examinations of multilingual experience, Mechelli et al. related the PGa to the "structural plasticity in the bilingual brain"^67^. More recently, Della Rosa et al.^68^ described it as "a neural interactive location for multilingual talent". Possibly the closest effect to the one we report in the present study comes from Lee et al.^69^, who associated its gray matter density with the number of learned words, but in English-speaking *monolingual* adolescents. Our measure of "Typological Diversity" weighs the lengths of experience with each of our participants' languages by the distance between them. This language distance measure is based on a normalized measure of overlap between languages' lexicons^27^. We therefore propose that the reported clusters of BOLD activity recorded during an English Word Match Task reflect a neural signature of lexical experience accumulated across different languages.

Lexical experience measures have similar neural underpinnings in mono-, bi- and multilingual samples. Are these anatomical and functional correlates a cause or a consequence of this specific behavioral phenotype? Since the 5-year-old participants in our study could be considered non-elective multilinguals (i.e., they did not consciously choose to learn new languages), and the Typological Diversity index is based on the amount of *environmental* exposure to the different languages, it seems unlikely that it is the brain structure and function that *causes* vast knowledge of vocabulary^69^. Rather, we see our results as a consequence of environmentally driven functional neuroplasticity that represents a neural signature of typologically diverse language environment. Language experience-induced plasticity in the IPL in longitudinal designs has indeed been shown by other studies^70^. Our data extend previously reported results and show that the degree of typological diversity (at the level of the lexicon) in multilingual language experience will also be reflected in how the brain processes L1 words. Of note, although the clusters reported in the analyses testing our Hypothesis 2 and 3 did not fully overlap, their localization was restricted to cytoarchitectonically the same area, PGa of the IPL. The differentiation between the precise functional roles of these two regions could be an area of future research. Taken together with prior evidence, our fMRI results characterize IPL PGa area as a signature of multilingual lexical richness. Furthermore, its activation levels reflect the total extent of lexical experience across *all* languages, with respect to typological overlaps and differences between those languages.

Our methodology provides a novel way to describe the continuum of multilingual language experience, including information on the relative similarities and differences between all languages a person has been exposed to during their life. Approaches acknowledging the parametric nature of multilingualism and going beyond a simple dichotomous classification of bi-/multilinguals *versus* monolinguals, are only starting to gain momentum in the field. Our "Typological Diversity" index can be used either as a variable of interest, or as a control variable in future studies with linguistically diverse populations, thus potentially contributing to more inclusive experimental cohorts. Of note, in the present study (for analyses testing Hypothesis 2), we excluded participants who had less than 50% of English exposure because the index (which is based on proportions of language exposure) would yield the same values for, e.g., two Cantonese—English bilingual individuals exposed to the two languages at the relative ratios of 0.4–0.6 and 0.6–0.4. While this constraint is necessary for a study with L1 vocabulary (or any other language-specific measure) as outcome variable, it is not an impediment when investigating outcomes not tied to a specific language, like e.g., executive functioning, attentional processes, etc. One particular strength of the proposed approach of quantifying multilingualism with Rao's quadratic entropy equation^25^ is its flexibility: it can be calculated with different measures of either language experience or typology. The cumulative lengths of exposure to every language could be exchanged by a proficiency index or age of acquisition measures, depending on the research questions at hand. Similarly, typological relations between languages were operationalized here with a lexical distance measure. Computing Typological Diversity indices for different domains of language opens exciting opportunities for probing the relationship between language experience, cognition, and the brain. For example, given the division of labor of the IPL in language tasks between semantics, phonology, and structural aspects of language^57^, Typological Diversity at the level of phonology, lexicon and morphosyntax, could have spatially diverging neural signatures in the parietal lobule. Another direction of future research could involve comparing the relative effects of Typological Diversity at different linguistic domains on executive functioning and cognitive control, thus potentially bringing new insights into the debated "bilingual advantage"^1^. Quantifying the multidimensional nature of multilingualism with cross-linguistically informed data will help future research investigate language learning, processing, and control in linguistically diverse populations with more precision.

Although informative and ecologically valid, our approach of quantifying the continuum of multilingual experience could be improved. First, our measure of language exposure does not account for languages present in participants' environment earlier *versus* later in life. Given the proposed "sensitive periods" for development of different aspects of language^71^ future work might distinguish between stages of language development. This might prove useful in further elucidating the role of typological linguistic diversity in language development. Furthermore, our measurement does not account for variation *within* the languages that children hear. Both quantity and quality of input are likely a "stable property of individual caregivers"^72^ that can influence children's language outcomes. Although we attempted to alleviate these effects by controlling for SES in all our analyses, such systematic variation is not completely determined by SES (even though it is indeed associated with it)^73^. Obtaining more detailed estimations of language behaviors of caregivers (e.g., ^73^) could help improve language exposure estimations, though in samples as linguistically diverse as ours, it would require unprecedented effort and resources. Lastly, our approach could be refined by varying typological features of participants' languages more systematically (see the "maximally diverse languages" approach for studying monolingual language acquisition^74^ and its strength in informing language development theory by linguistic typology). Indeed, the representation of lexical distance to the target language in our sample, especially in the sub-sample of participants whose L2 distance was related to their vocabulary scores and neural data (i.e., in models testing Hypothesis 3), was not uniform and could have skewed the obtained results. Future studies should therefore aim at replicating the language distance effect in samples where lexical distance to the target language is more uniformly distributed.

In conclusion, we show that typological linguistic diversity at the level of lexicon is associated with multilingual children's L1 knowledge and neural markers of L1 processing. The effects of typology of multilinguals' other languages are small but significant, and predict children's expressive vocabulary skills *above and beyond* cumulative length of exposure to the target language, cognitive, and demographic factors. Typological Diversity can also be associated with specific neural signatures in regions tied to neuroplasticity of bi- and multilingualism and lexical knowledge, thus refining our understanding of the neural underpinnings of language experience. The study also, for the first time, offers methodological tools for characterizing typological diversity of heterogeneous, multilingual samples.

## Supplementary Information

Supplementary Information 1.

## Data Availability

Public archiving of anonymized study data is not permitted by the corresponding ethics approval. Therefore, the datasets generated and analyzed during the current study are not publicly available but are available from the corresponding author on reasonable request and on the following conditions: approval from the local research ethics committee and with appropriate safeguards to protect from the identification of individuals as well as completion of a request form (https://osf.io/s5j42/files/osfstorage/6315a610db9397159d11eb39).
