# Correction: Language combinations of multilinguals are reflected in their first-language knowledge and processing

**DOI:** 10.1038/s41598-025-17838-w

**Published:** 2025-09-18

**Authors:** Olga Kepinska, Jocelyn Caballero, Myriam Oliver, Rebecca A. Marks, Stephanie L. Haft, Leo Zekelman, Ioulia Kovelman, Yuuko Uchikoshi, Fumiko Hoeft

**Affiliations:** 1 Department of Psychiatry and Behavioral Sciences, Weill Institute for Neurosciences, University of California, San Francisco, San Francisco, CA 94143 USA; 2 Department of Psychological Sciences, University of Connecticut, Storrs, CT 06269 USA; 3 Brain and Language Lab, Cognitive Science Hub, University of Vienna, 1090 Vienna, Austria; 4 Department of Cognition, Emotion, and Methods in Psychology, Faculty of Psychology, University of Vienna, 1010 Vienna, Austria; 5 Faculdad de Ciencias de la Salud, Universidad Europea de Valencia, 46010 Valencia, Spain; 6 Department of Psychology, University of Michigan, Ann Arbor, MI 48109 USA; 7 McGovern Institute for Brain Research, Massachusetts Institute of Technology, Cambridge, MA 02139 USA; 8 Department of Psychology, University of California Berkeley, Berkeley, CA 94704 USA; 9 Speech and Hearing Bioscience and Technology, Harvard University, Cambridge, MA USA; 10 School of Education, University of California, Davis, Davis, CA 95616 USA; 11 Brain Imaging Research Center, University of Connecticut, Storrs, CT 06269 USA; 12 Departments of Mathematics, Neuroscience, Psychiatry, Educational Psychology, Pediatrics, Computer Science and Engineering, University of Connecticut, Storrs, CT 06269 USA; 13 Haskins Laboratories, New Haven, CT 06511 USA

Correction to: *Scientific Reports* 10.1038/s41598-023-27952-2, published online 02 February 2023

The original version of this Article contained an error.

In the Methods section, under the subheading ‘Indices’, the Shannon’s entropy (*H*) equation was incorrectly given as:

$$H = - \mathop \sum \limits_{{{\text{i}} = 0}}^{{\text{n}}} p_{i} ~log_{2}\,i$$

The correct equation appears below.

$$H = - \mathop \sum \limits_{{{\text{i}} = 1}}^{{\text{n}}} p_{i} ~log_{2}\,p_{i}$$

The original Article has been corrected.

