# Supplementary material for: Language combinations of multilinguals are reflected in their first-language knowledge and processing
Source: Sci Rep. 2023 Feb 2;13:1947. doi: 10.1038/s41598-023-27952-2 (PMC9895446; doi:10.1038/s41598-023-27952-2)
Supplement: Supplementary file 1 — Supplementary Information 1. [file 41598_2023_27952_MOESM1_ESM.pdf]

Supplementary materials

Methods

Independent measures

Language Exposure data were collected using detailed parental questionnaires and in-person interviews. The length of cumulative exposure to any language each child in the sample came into contact with during their lives was calculated following methods described in[1] and using an adapted (from three to five languages) version of the 'Amount of language exposure in the past' section of the Bilingual Language Experience Calculator (BiLEC) form. The exact form used is available on the OSF profile of the current study[2]. The following information was used:

a. how many and which languages a child has been exposed to so far at home, (if applicable) at day-care and in any other structured way (e.g., through a baby-sitter or a play group). We include also relatively short language experiences ("1 hour a week for 6 months"), since even limited language exposure have been reported to have a significant impact on individual's cognitive outcomes[3,4];
b. what proportion of time each individual living in the house spoke any given language for each one-year period in the child's life?
c. if the child attended day-care, or other out-of-school activities, how much time did they spend there per week, and what was the approximate time ratio of languages used for instruction for each one-year period in the child's life;
d. time spent at school and language use at school quantified based on data available on applicable school district individual schools' websites.

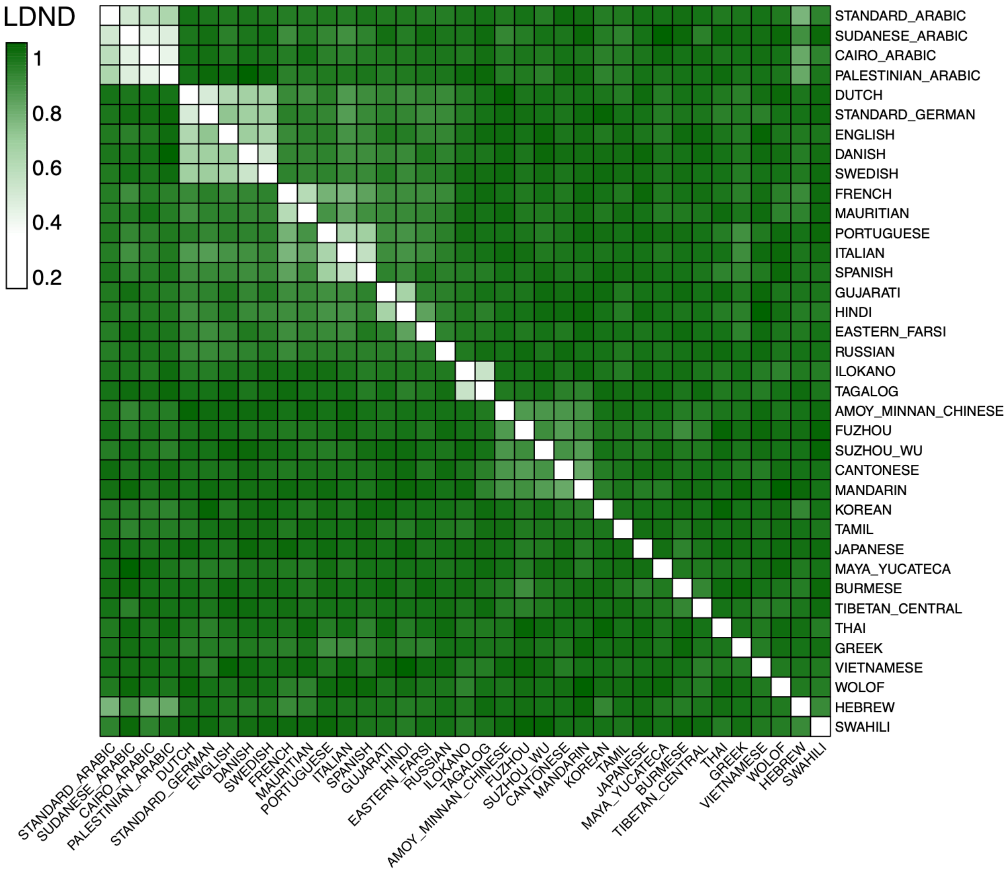

Figure S 1. Similarity matrix of lexical distances (operationalized with LDND) between all languages represented in our sample collected through the Automated Similarity Judgment Program (ASJP) Database. The figure was generated in R[5], with the package pheatmap[6], version 1.0.12.

LDND	Language
0	ENGLISH
0.6322	DUTCH
0.6626	SWEDISH
0.6962	DANISH
0.7213	STANDARD_GERMAN
0.8898	ITALIAN
0.9206	FRENCH
0.9225	SPANISH
0.9332	EASTERN_FARSI
0.9335	RUSSIAN
0.9454	MAURITIAN
0.9545	PORTUGUESE
0.9568	SUDANESE_ARABIC
0.9626	GREEK
0.9649	MAYA_YUCATECA
0.9704	HINDI
0.9741	CAIRO_ARABIC
0.9765	HEBREW
0.9803	STANDARD_ARABIC
0.9805	GUJARATI
0.9831	KOREAN
0.9852	CANTONESE
0.9875	SWAHILI
0.9896	ILOKANO
0.9928	WOLOF
0.9936	THAI
0.9939	JAPANESE
0.9994	FUZHOU
1.0081	TAMIL
1.0127	MANDARIN
1.0168	PALESTINIAN_ARABIC
1.018	TIBETAN_CENTRAL
1.0193	AMOY_MINNAN_CHINESE
1.0213	TAGALOG
1.023	BURMESE
1.0329	SUZHOU_WU
1.0406	VIETNAMESE

Table S 1. List of the lexical distances (LDND) of all languages represented in the current study to English.

	Diversity	Typological diversity
Exposure to English	-0.877	-0.899
Diversity	-	0.976

Table S 2. Correlation coefficients between the three language exposure indices included in the study.

Dependent measures

The fMRI task consisted of consecutive auditory presentation of two English words. Children were asked to indicate (with a button press) whether the words matched in form, i.e., whether they were the same or different. The task was presented in a 3.8 min functional run and consisted of 6 blocks separated by 12 s inter-block rest periods. 24 trials were included in each block, half of which were matching. Each 6 s trial consisted of Word 1, followed by Word 2 2000 ms later, followed by a 2000 ms question mark (see Figure S2). Participants heard two words and were asked to indicate via button press whether the two words were the same or not (e.g., “pants” – “pants” = yes, “hole” – “mop” = no). For each individual trial, stimuli were matched for the number of syllables and phonemes. All words had one or two syllables and were an average of 4.23 phonemes long.

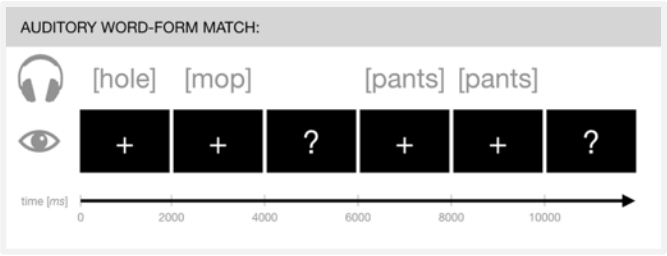

Figure S 2. In-scanner task: an example of 2 word pairs from the English Word Match Task.

Covariates of no-interest

Measure	Statistic	Raw score	Standardized score
**Independent measures:**			
Cumulative length of exposure to English	*M (SD)*	0.79 (0.21)	
	min	0.21	
	max	1.00	
Diversity of language exposure	*M (SD)*	0.43 (0.30)	
	min	0.00	
	max	1.35	
Typological diversity of language exposure	*M (SD)*	0.23 (0.17)	
	min	0.00	
	max	0.64	
**Dependent measures:**			
Receptive English vocabulary (PPVT)	*M (SD)*	116.60 (20.80)	100 (15)
	min	62	60.63
	max	158	129.85
Expressive English vocabulary (WJ-IV-PV)	*M (SD)*	24.95 (3.60)	100 (15)
	min	13	56.90
	max	33	134.28
**Covariates of no-interest:**			
age	*M (SD)*	5.68 (0.35)	
	min	5.05	
	max	6.41	
gender	*male:female ratio*	97:65	
SES	*Mdn (SD)*	17 (1.99)	
	min	10	
	max	21	
nonverbal reasoning (KBIT-2)	*M (SD)*	19.02 (5.52)	100 (15)
	min	8	70.04
	max	37	148.83
handedness	*right:left:ambidextrous ratio*	70:20:2	
d' score (fMRI task)	*M (SD)*	3.45 (1.50)	
	min	0.24	
	max	5.40	

Table S 3. Descriptive statistics for the independent and dependent measures, and covariates of no-interest in the overall sample; where applicable raw scores are accompanied by scores standardized for the current sample and centered around 100 with *SD* = 15.

Results

Hypothesis 1: cluster analysis

The two-stage cluster analysis consisted of (i) approximation of data grouping via a hierarchical cluster analysis (using Ward's minimum variance method and Euclidian distance as the similarity measure for the language assessments), and (ii) its refinement with a *k*-means cluster analysis. PPVT and WJ-IV-PV scores were first standardized (z-transformed) and a distance matrix between the observations was calculated using the R dist function, without the information about children's amount of exposure to English, and accounting for the control variables. According to the *k*-means cluster analysis, using the 'elbow' method, the data could be grouped in two clusters. The two-cluster solution classified participants into a "high-vocabulary" (N =74) and "low-vocabulary" (N = 86) groups, whose characteristics are summarized in Table S4 and Figure S3. While a low proportion of exposure to English was more prevalent in the group with low vocabulary scores, a exposure to English higher than 60% was not exclusive to the "high-vocabulary" group, as was predominantly the case in [7]. In fact, the majority of "low-vocabulary" participants were exposed to English equal to or longer than 60% of their lives. This is not to say that Exposure to English did not differentiate between the groups (a two sample t-test on the proportion of cumulative length of exposure to English was significant: $t(158) = 4.81$, $p < .001$). Rather, for our dataset, the English monolingual-like performance on vocabulary tests might be achieved at a different level of proportion of exposure to English than the previously reported 60%; another possibility is that vocabulary knowledge and length of exposure to L1 in kindergartners is fully linear, and that there is no threshold of minimal exposure length above which the vocabulary scores would not increase further. We test this possibility in subsequent sections.

		Cluster 1 (high vocabulary)		Cluster 2 (low vocabulary)	
		raw	group-standard	raw	group-standard
PPVT	*M*	130.89	111.75	104.60	89.89
	min	104.00	94.35	62.00	54.92
	max	158.00	141.99	142.00	108.31
WJ-IV-PV	*M*	27.59	111.81	22.81	89.84
	min	22.00	95.22	15.00	56.65
	max	33.00	141.29	29.00	116.70
Exposure to English	*M*	87%		72%	
	min	34%		21%	
	max	100%		100%	
# monolinguals		7		3	
# multilinguals with ≥ 60% English exposure		62		55	
# multilinguals with < 60% English exposure		5		28	

Table S 4. Summary of participant grouping according to the *k*-means cluster analysis. The "group-standard" scores are included for reference. They were computed by creating residual values controlled for age, gender, SES and non-verbal intelligence, transformed into *z*-scores and centered at a value of 100 with *SD* = 15.

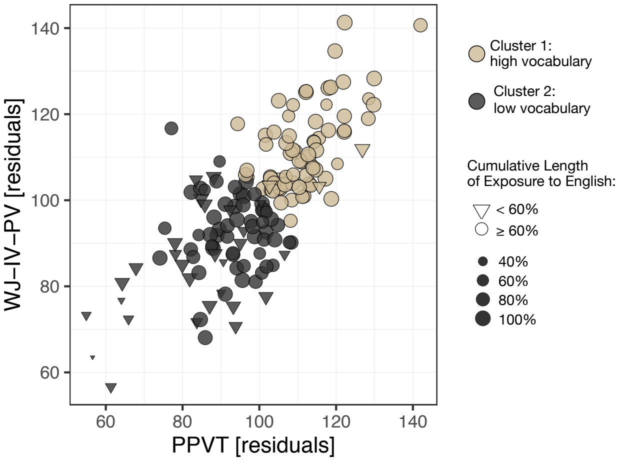

Figure S 3. Receptive (PPVT) and expressive (WJ-IV-PV) English vocabulary scores as classified into two clusters according to the *k*-means cluster analysis. We used residual values of PPVT and WJ-IV-PV, controlling for age, gender, SES and non-verbal intelligence. Data were standardized for the analysis and as a point of reference, centered around 100 with *SD* = 15 for visualizations. Triangles denote participants with Cumulative Length of Exposure to English lower than 60%, dots – equal or higher than 60%, the size of each represents the proportion of Cumulative Length of Exposure to English of a participant.

Hypothesis 1: curve-fitting analyses

To establish whether the relationship between vocabulary knowledge and Exposure to English in our sample was fully linear, we further performed curve-fitting analyses, first estimating linear, quadratic and cubic relationships between the dependent and independent variables [cf. 8] and controlling for covariates (age, gender, SES and nonverbal reasoning skills), see Table S 5. For both receptive and expressive vocabulary scores, the linear plus quadratic models accounted for more variance than the linear models alone, but the additional unique variance explained by the quadratic models was in both cases lower than 1%. Bayes Factor for the quadratic versus linear models indicated that in both cases there was more evidence in favor of the linear fits. The linear plus quadratic plus cubic models for both dependent variables resulted in slightly worse fits and Bayes Factors indicating very strong evidence in favor of the quadratic models. To further adjudicate on the linear versus nonlinear fits, with the mgcv R package[9–12] we used general additive models (GAMs), which determine the optimal shape of potential non-linearity automatically. We fit GAMs to the PPVT and WJ-IV-PV scores, incorporating a smooth term for the Exposure to English variable, controlling for covariates of no-interest and comparing the general additive models to the linear ones. As shown in Table S5,

adding a smooth term for Exposure to English did not lead to significantly improved fits over the linear models, neither for PPVT nor for WJ-IV-PV.

	ΔR^2	*F*	*DF*	*edf*	*p*	BIC	BF_{10}
**Receptive vocabulary ~ Exposure to English:**							
Linear	0.156	20.62	5,156	-	< .001	1395.533	10029896.000
Quadratic	0.009	17.96	6,155	-	< .001	1397.299	0.414
Cubic	-0.003	15.33	7,154	-	< .001	1402.236	0.085
GAM [*vs.* Linear]	-0.011	19.39	-	1.768	< .001	1396.577	0.593
GAM [*vs.* Quadratic]	< -0.001						1.435
**Expressive vocabulary ~ Exposure to English:**							
Linear	0.193	13.74	5,154	-	< .001	829.478	26166939.000
Quadratic	0.002	11.71	6,153	-	< .001	833.108	0.163
Cubic	-0.002	10.08	7,152	-	< .001	837.612	0.105
GAM [*vs.* Linear]	0.002	27.95	-	1.371	< .001	830.430	0.621
GAM [*vs.* Quadratic]	< -0.001						3.815

Table S 5. Multiple regression model parameters for the linear, quadratic and cubic relationships between English receptive vocabulary (PPVT) and English expressive vocabulary (WJ-IV-PV), and the proportion of Cumulative Length of Exposure to English. The models were compared in an incremental fashion, i.e., the linear model was compared to a baseline model containing the covariates of no-interest only, the quadratic model to the linear, and the cubic to the quadratic.

Hypothesis 1: breakpoint discovery procedure

Finally, to see whether there were different linear relationships in our data, we employed a breakpoint discovery procedure [cf. 13] using the segmented R package[14]. Breakpoint discovery allows to estimate the existence and location of a possible change in the relationship between variables by fitting a set of regression models, each with a breakpoint at different level of the independent variable. The number of breakpoints of the segmented relationship was selected according to the BIC criterion. Both for the receptive and expressive vocabulary scores, a no-breakpoint model had the lowest BIC, and thus the best fit (1395.533 *versus* 1401.897, for a one breakpoint model, and 1404.523 for a two breakpoints model in case of PPVT; 829.478 *versus* 837.140, for a one breakpoint model, and 843.395 for a two breakpoints model in case of WJ-IV-PV).

Hypothesis 2: neuroimaging data

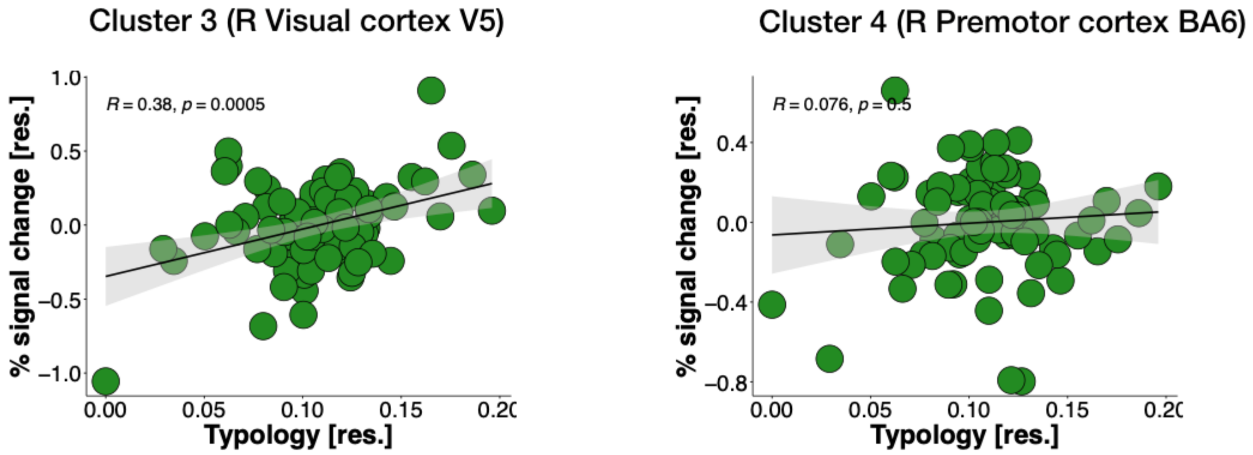

Figure S 4. Percentage signal change values for the BOLD signal recorded during the English Word Match Task in Clusters 3 and 4 (R Visual cortex V5, and the R Premotor cortex BA6, respectively), as a function of residual values of Typology (i.e., Typological Diversity index controlled for covariates of no-interest and length of exposure to all languages).

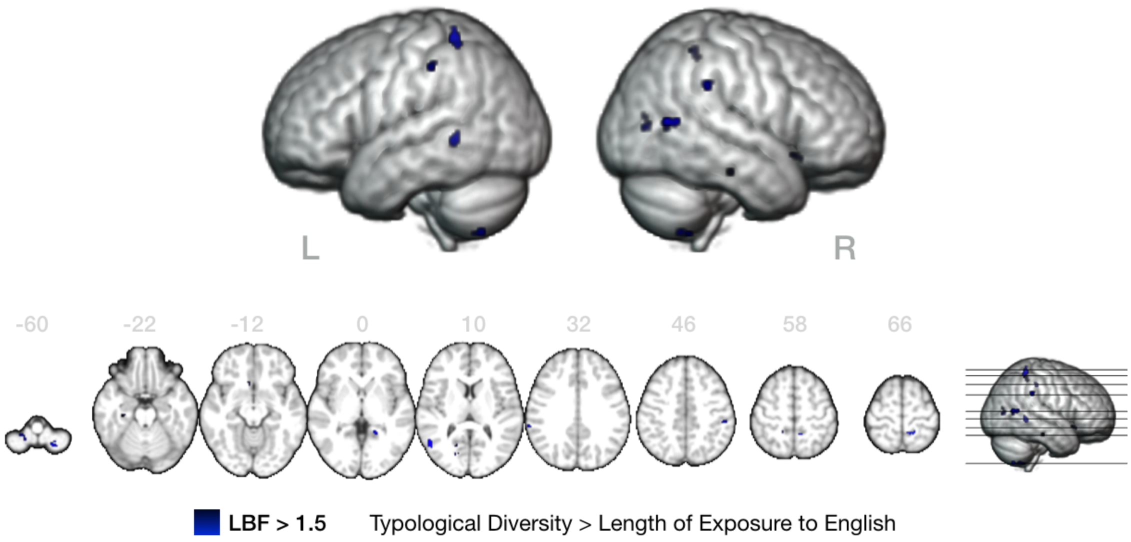

Figure S 5. Clusters of voxels (in blue) showing preference for the model including typological information on participants' language background ("Typological Diversity") over a model accounting for Length of Exposure to English only.

Hypothesis 3

We performed an exploratory comparison between bilingual participants exposed to two most frequently represented L2s in our data base, differing in their distance to English: Spanish, LDND = 0.9225 (n = 76) and Cantonese, LDND = 0.9852 (n = 22). Non-parametric Welch's t-test was performed given the unequal size of groups. Although the Cantonese-exposed children had on average lower scores for both receptive and expressive vocabulary tests that the Spanish-exposed ones (PPVT: $M_{Cantonese}$ = 113.82, SD = 19.88, and $M_{Spanish}$ = 119.68, SD = 18.13, and WJ-IV-PV: $M_{Cantonese}$ = 24.5, SD = 4.10, and $M_{Spanish}$ = 25.53, SD = 3.00), the difference was not statistically significant ($t(30.99) = -1.42$, p = .16, and $t(27.42) = -0.41$, p = .68), for PPVT and WJ IV PV, respectively), see Figure S6 (B) and (D). This analysis showed that the difference in lexical similarity to English of Spanish and Cantonese might not be enough to be significantly related to children's English vocabulary. We speculate that the effect of L2 Distance to English on children's English vocabulary knowledge might be more pronounced when comparing L2s with LDND spanning a wider range than 0.9225 - 0.9852 (like in case of Spanish and Cantonese respectively). However, due to a skewed distribution of LDND of L2s in the current sample, we are left to propose that this speculation be tested in future studies varying lexical distance to the target language more systematically, i.e., including languages lexically overlapping with the target language very little (e.g., Cantonese-English, LDND = 0.9852), and overlapping more (like e.g., Dutch-English, LDND = 0.6322, or Swedish-English, LDND = 0.5292).

To probe the effect of L2 Distance in the neuroimaging data, we, again, performed a series of exploratory comparisons between data from bilingual participants exposed to Spanish (n = 38) and Cantonese (n = 14), see violin plots in Figure S7, panel A. In all investigated clusters, the percentage BOLD signal change values were lower for Cantonese participants; the difference was significant in clusters (2) and (4) according to a non-parametric Welch t-test.

The Interaction model ($H3\text{-}m_3$) was preferred over the main effects only model ($H3\text{-}m_2$) in 17 clusters (Figure S7, panel B, Table S7). To gain further insight into the directionality of this effect, we performed a series of ROI analyses in the 5 largest clusters. In the largest cluster (in the right Cerebellum IX), for participants with an L2 most distant to English, the relationship between length of Exposure to English and percentage BOLD signal change values was positive (i.e., the longer their Exposure to English, the higher the brain activity); participants with an L2 closer to English showed a negative relationship between BOLD response and Exposure to English. In the remainder of the investigated clusters, the moderating effect of L2 Distance on BOLD signal – Exposure to English relationship, was the opposite. Participants with more distant languages showed a negative BOLD – Exposure to English relationship; for those with an L2 closer to English, the more time they were exposed to English, the higher their brain activity.

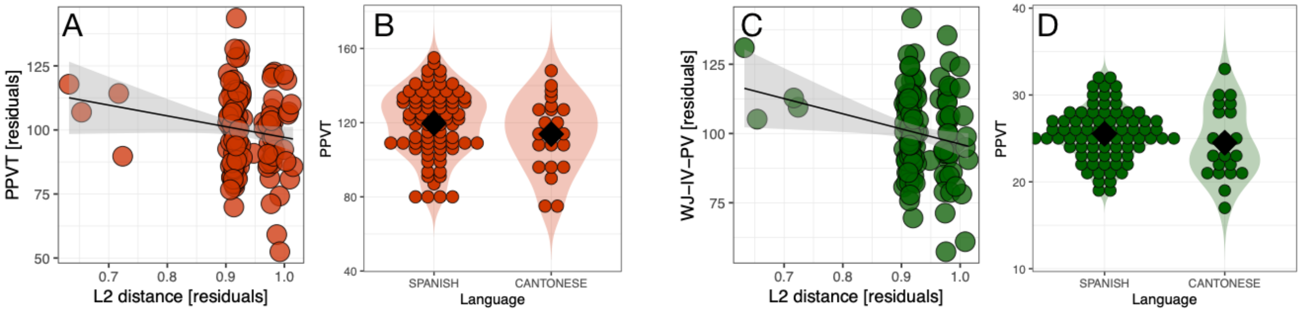

Figure S 6. English receptive (PPVT) and expressive vocabulary (WJ-IV-PV) scores of a bilingual sub-sample of participants, as a function of their L2's lexical distance to English. Figures A and C show all bilinguals, while figures B and D, a matched selection of participants exposed to two most frequently represented L2s in our data base: Spanish and Cantonese.

	$H3\text{-}m_0 >$	$H3\text{-}m_1 >$	$H3\text{-}m_2 >$	$H3\text{-}m_3 >$
$H3\text{-}m_0$		875	1119	693
$H3\text{-}m_1$	10860		1147	732
$H3\text{-}m_2$	65831	18983		669
$H3\text{-}m_3$	124528	89830	19322	

Table S 6. Number of voxels showing model preference according to the LBF > 3 threshold for each of the performed model comparisons testing Hypothesis 3.

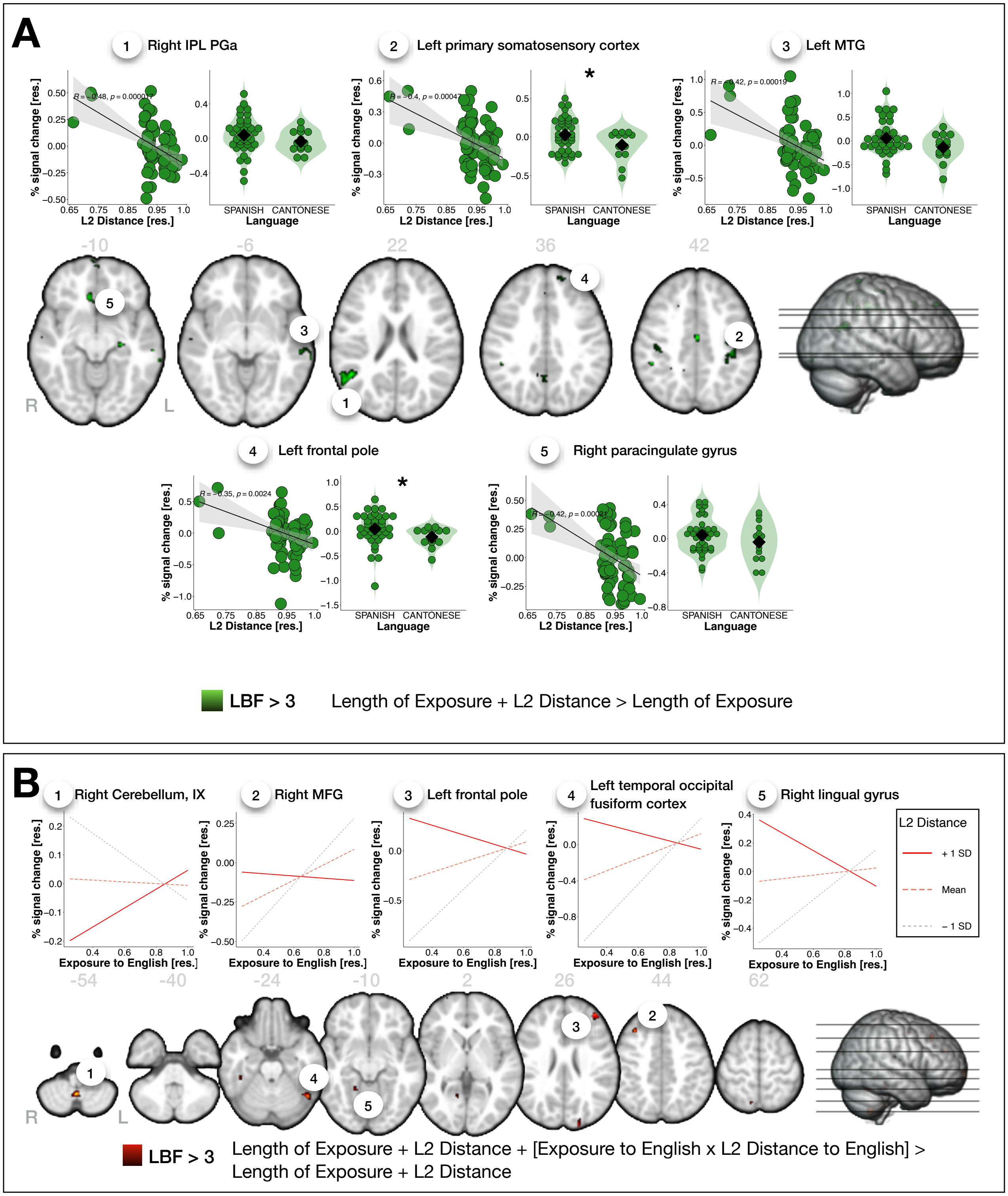

Figure S 7. (A) Effect of L2 Distance to English in the bilingual sub-sample of participants (n = 73). Shown on the brain slices are clusters of voxels (in green) showing preference for the model including the L2 Distance to English variable over a model accounting for cumulative length of exposure to English. The scatter plots, present percentage signal change values (residuals) in the five biggest identified clusters as a function of residual values (i.e., controlled for covariates of no-interest and length of exposure to English) of L2 distance to English. Violin plots illustrate percentage signal change values in the clusters in question for the two largest L2 groups: English-Spanish and English-Cantonese bilinguals. (B) The interaction effect between the Length of Exposure to English variable and the L2 Distance variable in the bilingual sub-sample of participants. Bottom panel (brain slices) presents clusters of voxels (in red) showing preference for the model including the interaction between the Length of Exposure to English and L2 Distance to English variables over a model accounting for both variables independently. Presented in the top panel are percentage signal change values (residuals) in the five biggest identified clusters as a function of residual values (i.e., controlled for covariates of no-interest) of Exposure to English for different levels of the L2 distance to English variable.

Cortical region	L/R	size (voxels)	LBF_{max}	peak location (voxels)		
				x	y	z
(*H3-m$_2$*) Exposure to English + L2 Distance to English > (*H3-m$_1$*) Exposure to English						
1. Inferior parietal lobule PGa	R	165	10.9	20	36	48
2. Primary somatosensory cortex BA2	L	114	9.18	65	50	58
3. Middle temporal gyrus, posterior division	L	53	6.07	75	50	32
4. Frontal pole	L	47	6.33	51	92	56
5. Paracingulate gyrus	R	39	7.32	41	79	31
6. Premotor cortex BA6	R	37	6.11	25	57	68
7. Inferior parietal lobule PFm	R	36	9.04	20	44	58
8. Primary motor cortex BA4p	L	34	6.94	61	53	58
9. Superior frontal gyrus	R	33	6.47	35	74	59
10. Superior parietal lobule	R	31	6.96	32	38	61
11. Postcentral gyrus	R	31	5.19	23	52	56
12. Superior parietal lobule	L	30	5.39	59	42	70
13. Frontal pole	R	28	5.91	44	97	29
14. Frontal pole	R	27	8.66	34	87	60
15. Postcentral gyrus	R	26	9.86	32	44	73
16. Middle temporal gyrus, posterior division	R	26	4.65	15	57	29
17. Cingulate gyrus, anterior division	L	25	8.85	45	57	57
18. Precentral gyrus	L	24	4.46	60	57	65
19. Precuneous cortex	R	23	4.54	40	36	55
20. Middle temporal gyrus, posterior division	L	19	5.45	78	45	36
21. Inferior temporal gyrus, anterior division	R	14	4.3	22	61	13
22. Inferior parietal lobule PFm	R	14	3.91	20	40	62
23. Amygdala	L	14	5.12	57	60	20
24. Visual cortex V3V	R	14	8.25	26	17	29
25. Hippocampus entorhinal cortex	L	12	7.09	58	55	21
26. Anterior intra-parietal sulcus hIP1	L	11	4.84	65	39	59
27. Paracingulate Gyrus	R	11	5.18	37	85	39
28. Middle temporal gyrus, posterior division	R	10	4.4	13	50	27
29. Hippocampus (cornu ammonis)	L	10	9.28	58	53	31
30. Paracingulate gyrus	R	10	3.83	43	83	51
(*H3-m$_3$*) Exposure to English X L2 Distance to English > (*H3-m$_2$*) Exposure to English + L2 Distance to English						
1. Cerebellum, IX	R	78	9.75	44	35	9
2. Middle frontal gyrus	R	47	8.02	27	77	57
3. Frontal pole	L	44	10.2	67	85	50
4. Temporal occipital fusiform cortex	L	43	10.4	67	35	23
5. Lingual gyrus	R	41	5.26	39	33	38
6. Inferior temporal gyrus, posterior division	L	33	6.8	77	50	23
7. Frontal pole	R	24	11.6	34	99	34
8. Occipital pole	L	21	6.01	54	15	49
9. Lingual gyrus	R	20	4.78	35	39	32
10. Intracalcarine cortex	L	19	4.39	51	21	38
11. Temporal fusiform cortex, posterior division	R	16	7.53	25	46	25
12. Inferior temporal gyrus, posterior division	R	15	4.35	17	47	27
13. Precentral gyrus	R	14	5.64	25	61	61
14. Superior parietal lobule 7A	R	13	4.84	39	29	68
15. Middle frontal gyrus	L	11	4.95	57	66	63
16. Frontal pole	L	10	7.53	58	96	45
17. Precentral gyrus	R	10	4.06	30	62	63

Table S 7. Brain areas showing model preference for a model including Length of Exposure to English and L2 Distance to English above a Length of Exposure to English model only, according to LBF > 3 (and with clusters consisting of at least 10 voxels, ordered by size). The x, y and z coordinates are in the age-appropriate pediatric MNI NIHPD space [15], the regions were labelled according to Harvard-Oxford Cortical and Sub-cortical Structural Atlases, Jülich Histological Atlas and Cerebellar Atlas (all implemented within FSLeyes, part of FSL, after non-linearly transforming the atlases to the pediatric space).

References

1. Unsworth, S. Assessing the role of current and cumulative exposure in simultaneous bilingual acquisition: The case of Dutch gender. *Biling. Lang. Cogn.* **16**, 86–110 (2013).
2. Kepinska, O. & Hoeft, F. *Effects of linguistic diversity on L1 lexical processing and vocabulary development in kindergartners.* (2020).
3. Bice, K. & Kroll, J. F. English only? Monolinguals in linguistically diverse contexts have an edge in language learning. *Brain Lang.* **196**, (2019).
4. Knightly, L. M., Jun, S.-A., Oh, J. S. & Au, T. K. Production benefits of childhood overhearing. *J. Acoust. Soc. Am.* **114**, 465–474 (2003).
5. R Development Core Team. R: A Language and Environment for Statistical Computing. *R Foundation for Statistical Computing* **1**, 409 (2015).
6. Kolde, R. pheatmap: Pretty Heatmaps. (2019).
7. Cattani, A. *et al.* How much exposure to English is necessary for a bilingual toddler to perform like a monolingual peer in language tests? *Int. J. Lang. Commun. Disord.* **49**, 649–671 (2014).
8. Unsworth, S., Chondrogianni, V. & Skarabela, B. Experiential measures can be used as a proxy for language dominance in bilingual language acquisition research. *Front. Psychol.* **9**, 1–15 (2018).
9. Wood, S. N. Fast stable restricted maximum likelihood and marginal likelihood estimation of semiparametric generalized linear models. *J. R. Stat. Soc.* **73**, 3–36 (2011).
10. Wood, S. N. *Generalized additive models: An introduction with R (second edition)*. (Chapman and Hall, CRC Press., 2017).
11. Wood, S. N. Stable and efficient multiple smoothing parameter estimation for generalized additive models. *J. Am. Stat. Assoc.* **99**, 673–686 (2004).
12. Wood, S. N., N., Pya & Safken, B. Smoothing parameter and model selection for general smooth models (with discussion). *J. Am. Stat. Assoc.* **111**, 1548–1575 (2016).
13. Veríssimo, J., Verhaeghen, P., Goldman, N., Weinstein, M. & Ullman, M. T. Evidence that ageing yields improvements as well as declines across attention and executive functions. *Nat. Hum. Behav.* (2021). doi:10.1038/s41562-021-01169-7
14. Muggeo, V. Selecting number of breakpoints in segmented regression: implementation in the R package segmented. (2020).
15. Fonov, V. *et al.* Unbiased average age-appropriate atlases for pediatric studies. *Neuroimage* **54**, 313–327 (2011).
